# When minds and networks matter: how mental health and social capital shape social frailty in older adults

**DOI:** 10.3389/fmed.2026.1778410

**Published:** 2026-05-28

**Authors:** Sarah Almutairi, Mohamed Hussein Ramadan Atta, Ibrahim Alasqah, Mona Metwally El-Sayed, Safia Gomaa Mohammed, Ahmed Abdellah Othman, Haitham Mokhtar Mohamed Abdallah, Shaimaa Mohamed Amin

**Affiliations:** 1 Department of Community, Psychiatric, and Mental Health Nursing, College of Nursing, Qassim University, Buraydah, Saudi Arabia; 2 Nursing Services Department, Qassim University Medical City, Buraydah, Saudi Arabia; 3 Nursing Department, College of Applied Medical Sciences, Prince Sattam Bin Abdulaziz University, Wadi Addawasir, Saudi Arabia; 4 Psychiatric and Mental Health Nursing Department, Faculty of Nursing, Alexandria University, Alexandria, Egypt; 5 School of Health, University of New England, Armidale, NSW, Australia; 6 Gerontological Nursing, Faculty of Nursing, Zagazig University, Zagazig, Egypt; 7 Nursing Administration, Faculty of Nursing, Sohag University, Sohag, Egypt; 8 Critical Care and Emergency Nursing, Faculty of Nursing, Alexandria University, Alexandria, Egypt; 9 Medical Surgical Department, College of Nursing, Jouf University, Sakaka, Saudi Arabia; 10 Community Health Nursing Department, Faculty of Nursing, Damanhour University, Damanhour, Egypt

**Keywords:** aging, geriatric assessment, mental health, older adults, social capital, social frailty

## Abstract

**Background:**

Social frailty represents a critical dimension of aging, yet its specific associations with mental health and social capital remain underexplored, particularly in non-Western contexts. This study investigated the influence of psychological distress and social capital on social frailty among older adults in Egypt.

**Methods:**

In a cross-sectional study conducted at a university hospital outpatient clinic, 316 adults aged ≥60 years were assessed using the Depression Anxiety Stress Scale-21 (DASS-21), the Personal Social Capital Scale-16 (PSCS-16), and the Social Frailty Scale (SFS-8). Data were analyzed using descriptive statistics, correlation analyses, and multiple linear regression.

**Results:**

Participants demonstrated moderate to high levels of social frailty (Mean = 5.55 ± 1.44). A significant positive correlation was found between psychological distress and social frailty (*β* = 0.333, *p* < 0.001), while a significant negative correlation was observed between social capital and social frailty (*r* = −0.349, *p* < 0.001). Multiple regression analysis confirmed that both higher psychological distress (*β* = 0.369, *p* < 0.001) and lower social capital (*β* = −0.327, *p* < 0.001) were significant independent predictors of social frailty, after controlling for key sociodemographic and health-related covariates. The final model explained 13.5% of the variance in social frailty scores (*F* = 24.510, *p* < 0.001).

**Conclusion:**

Psychological distress and low social capital are significant, independent contributors to social frailty in Egyptian older adults. These findings advocate for integrated geriatric care interventions that concurrently address mental well-being and strengthen social networks to effectively prevent and mitigate the adverse outcomes associated with social frailty.

## Introduction

The global population is aging rapidly. As of 2020, approximately 1 billion people were aged 60 years and older, and this number is projected to rise to 1.4 billion by 2030, meaning that one in six people worldwide will be in this age group. By 2050, the number of older adults is expected to double to 2.1 billion, while the population aged 80 years and above is projected to triple to approximately 426 million (1). Although many older adults remain active contributors to society as caregivers, volunteers, and workers, advancing age is also associated with increased vulnerability to a range of health challenges that may threaten well-being and independence in later life.

In response to population aging, the World Health Organization introduced the concept of healthy aging, defined as the ability to maintain and enhance the functional abilities that support well-being in older age (2). Despite this goal, aging is often accompanied by frailty, a clinical state characterized by reduced physiological reserves and diminished resilience to internal or external stressors due to cumulative declines across multiple body systems (3, 4). Frailty increases susceptibility to adverse outcomes such as falls, disability, illness, injuries, and mortality, especially when older adults encounter physical or psychological stressors (3, 5–8). Because of its substantial consequences for individuals and healthcare systems, frailty has become a major public health concern in aging societies (9).

Although frailty was initially conceptualized primarily in physical terms, it is now increasingly recognized as a multidimensional construct that also includes psychological and social domains (10, 11). Within this broader perspective, social frailty has emerged as an important but still underexplored subtype of frailty (12, 13). Social frailty refers to a decline in the social resources, social behaviors, and capacities needed to meet essential social needs (14–17). It includes reduced social participation, weakened social networks, limited social support, and difficulties in fulfilling basic social roles and expectations. Drawing on Social Production Functions Theory, Bunt et al. (18) defined social frailty as the persistent absence of one or more resources required to meet fundamental social needs and emphasized the importance of social behavior, engagement in activities, and self-management capacities in this process (18, 19).

Like physical frailty, social frailty is associated with adverse health outcomes and poses important challenges for health and social care systems. Evidence suggests that demographic characteristics, lifestyle factors, and health indicators may influence frailty across its physical, psychological, and social dimensions (20). Shared risk factors for physical and social frailty include female sex, low educational attainment, chronic illness, poor nutrition, and low levels of physical activity. Moreover, the coexistence of physical, psychological, and social frailty appears to confer a greater risk of disability and mortality than impairment in any single domain alone (21, 22). These findings highlight the need to understand social frailty not as an isolated social issue, but as an integral component of vulnerability in later life.

Mental health is one factor that may contribute meaningfully to this vulnerability. Older adults experience a substantial burden of mental health problems, particularly depression, anxiety, and psychological distress. Approximately 14% of individuals aged 60 years and older live with a mental disorder, accounting for 10.6% of years lived with disability in this age group, and more than one-quarter of global suicide deaths occur among older adults (23). Mental well-being in later life is shaped by accumulated life experiences and age-related stressors, including declining functional capacity, bereavement, retirement, income reduction, and social devaluation through ageism (24). In addition, physical problems such as chronic pain, frailty, mobility limitations, and dementia frequently coexist with mental health difficulties and may further increase the need for long-term care (25, 26).

A plausible conceptual pathway links mental health conditions to social frailty. Depression, anxiety, and psychological distress may reduce motivation, energy, confidence, and perceived self-worth, thereby limiting social participation and diminishing the ability to maintain meaningful social roles and relationships. At the same time, mental health problems may intensify withdrawal from social activities, decrease engagement with supportive networks, and impair self-management capacities, all of which are central features of social frailty. This relationship may be further reinforced by adverse social circumstances commonly experienced in later life. Social isolation and loneliness affect nearly one-quarter of older adults and are recognized as risk factors for mental disorders (24). Abuse, neglect, caregiving burden, poverty, chronic disease, residence in remote areas, and substance use disorders may also worsen psychological well-being and increase vulnerability among older adults (27–30). Accordingly, mental health conditions may not only coexist with social frailty but may also contribute to its development and persistence.

Social capital broadly refers to the resources embedded within social relationships and social structures that individuals can access through interaction with family, friends, neighbors, communities, and wider society (31–33). Conceptually, social capital may be understood in several complementary ways. Bonding social capital refers to resources available within close, homogeneous ties, such as family members and intimate friends, whereas bridging social capital refers to resources derived from more distant or heterogeneous social connections that may broaden access to information and opportunities. Social capital may also be distinguished into cognitive and structural forms. Cognitive social capital includes subjective elements such as trust, reciprocity, and sense of belonging, whereas structural social capital reflects observable aspects such as social participation, civic engagement, and the size or frequency of social contacts. These distinctions are important because different dimensions of social capital may influence later-life outcomes through different mechanisms.

A growing body of research has linked social capital to the health and well-being of older adults (31–35). Several studies have examined its association with loneliness. In Western Finland, lower levels of social capital, assessed through social contacts, participation, trust, and sense of belonging, were associated with loneliness, although only low trust remained significant among the oldest participants (35). Across 14 European countries, greater social capital, including more frequent social participation, was associated with lower loneliness, especially among older adults from low-income households (36, 37). In rural China, both bonding and bridging social capital were associated with loneliness among widowed older adults (38). Similarly, an intervention study in Spain showed that stronger social capital, particularly in the form of participation and support, reduced the likelihood of loneliness (31). Nevertheless, prior studies have used diverse conceptual and measurement frameworks, resulting in inconsistent findings and highlighting the need for more standardized and theoretically grounded approaches.

Loneliness in later life is shaped by multiple factors, including diminished social capital (11, 17, 20, 31, 39). However, although these relationships have been examined individually, less is known about how social capital and mental health operate together in shaping social frailty. Existing research suggests that social capital may protect against frailty and mental health problems among older adults. For example, Hu et al. (40) found that higher cognitive social capital was associated with a lower risk of physical frailty, with health-promoting lifestyles partially mediating this relationship (40). Likewise, Wang et al. (41) reported that social capital may reduce frailty risk through its effect on depressive symptoms (41). However, these studies primarily focused on physical frailty and did not adequately address social frailty as a distinct and multidimensional outcome.

Given that social frailty encompasses deficits in social resources, participation, and support, understanding the joint contribution of mental health and social capital is particularly important. Social frailty may precede, accompany, or exacerbate physical decline, thereby increasing the risk of adverse outcomes in older adults. Yet the interrelationship among mental health, social capital, and social frailty remains insufficiently explored. Therefore, the present study aims to address this gap by examining how mental health and social capital jointly influence social frailty in older adults. Clarifying these associations may contribute to the development of more integrative interventions to promote healthy, socially connected, and resilient aging.

## Subjects and methods

### Study design and setting

The study utilized a cross-sectional descriptive research design and adhered to the Strengthening the Reporting of Observational Studies in Epidemiology (STROBE) guidelines to ensure methodological rigor and transparency. It was conducted at the outpatient clinics of Zagazig University Hospitals, situated in Sharkia Governorate, Egypt. These hospitals serve as a leading tertiary care center in the region, providing healthcare services to a diverse population from both urban and rural areas of Sharkia and neighboring governorates. Renowned for offering a broad spectrum of specialized medical services, Zagazig University Hospitals are a cornerstone of comprehensive healthcare in the region. The outpatient clinics, in particular, are well-equipped to address a variety of medical conditions, including those frequently encountered in older adults.

### Sample size and study population

The study included older adults aged 60 years and above who attended the outpatient clinics at Zagazig University Hospitals during the data collection period. Participants were required to be able to communicate effectively and provide informed consent to participate in the study. However, the study excluded those with severe cognitive impairments or diagnosed dementia, as these conditions could compromise their ability to respond accurately to the study measures. Additionally, patients with acute medical conditions requiring immediate attention and those unwilling or unable to provide informed consent were excluded.

The sample size was determined using G*Power 3.1.9.7 software (42), with calculation parameters set at a power of 0.95, an alpha level of 0.001, and a moderate effect size of 0.15. The selection of a medium effect size (f^2^ = 0.15) for the sample size calculation was based on Cohen’s conventions for multiple regression analysis (43). In the absence of prior local studies directly examining the relationship between mental health, social capital, and social frailty in older adults, a medium effect size was considered the most appropriate and conservative estimate to ensure adequate statistical power. This approach is commonly used in similar psychosocial and gerontological studies when empirical effect size estimates are not available. Therefore, the assumption of a medium effect size was theoretically justified rather than derived from a specific prior study. Based on these criteria, a minimum of 304 participants was required. To account for potential dropouts, the target sample size increased to 330. Ultimately, 316 valid questionnaires were collected and included in the final analysis, resulting in a high response rate of 95.7%.

Eligibility criteria for exclusion of participants: Participants were excluded from the study if they met any of the following criteria: Presence of severe cognitive impairment or a documented diagnosis of dementia that could interfere with understanding the questionnaire or providing reliable responses. Acute medical conditions require urgent or immediate medical intervention during the data collection period. Severe communication difficulties (e.g., speech or hearing impairments) that prevent effective participation in the interview or questionnaire completion. Refusal or inability to provide informed consent to participate in the study. These exclusion criteria were applied to ensure the validity and reliability of the collected data and to maintain the ethical integrity of the research by protecting individuals who were not capable of participating meaningfully.

The researchers employed convenience sampling to recruit participants based on predefined inclusion criteria. Initially, 330 older adults were invited to participate in the study. However, 10 individuals declined the invitation, and four did not meet the eligibility criteria. Consequently, the final sample comprised 316 participants, whose responses were included in the analysis.

## Instruments

### The demographic form

The demographic form collected comprehensive information about participants, including their age, sex, marital status, educational attainment, and place of residence. It also captured details regarding their occupation and income level. To assess health-related behaviors, the form inquired about cigarette smoking and participation in regular physical exercise. Furthermore, it gathered data on family structure, the presence of chronic illnesses, the specific type of chronic disease, and the duration since diagnosis, offering valuable insight into the participants’ health profiles.

### Depression Anxiety Stress Scale-21 (DASS-21)

The Arabic version of the Depression Anxiety Stress Scale-21 (DASS-21), translated by Ali and Green (44) for use among Egyptian clients, is a validated tool designed to assess the distinct dimensions of depression, anxiety, and stress experienced over the past week (44). This version preserves the original structure of DASS-21, comprising 21 items evenly distributed across three subscales (45). Each subscale includes seven items rated on a four-point Likert scale, ranging from 0 (“Did not apply to me at all”) to 3 (“Applied to me very much or most of the time”). Higher scores reflect greater psychological distress. In the current study, the Arabic version demonstrated exceptional internal consistency, with a Cronbach’s alpha of 0.98.

### The Social Frailty Scale (SFS-8)

The Social Frailty Scale (SFS-8) is a specialized tool developed by Pak et al. in Singapore in 2020 to evaluate the social aspects of frailty among older adults (46). It encompasses eight items across three dimensions: social resources (e.g., meeting with friends, seeking advice, and trust), social activities and financial resources (e.g., going out, dining, and financial status), and fulfilling social needs (e.g., loneliness and conversations). Each item is scored as 0 or 1, resulting in a total score ranging from 0 to 8. Based on the total score, individuals are classified into one of three categories: non-frail (0–1), pre-frail (2–3), and frail (4–8). In the study by Zare et al. (17), the SFS-8 was cross-culturally adapted for Iranian older adults, with its psychometric properties rigorously evaluated (17). The scale demonstrated strong reliability, with a Cronbach’s alpha of 0.87, indicating high internal consistency. Construct validity was established through exploratory and confirmatory factor analyses, revealing factor loadings between 0.65 and 0.88 across the domains. In the current study, the Cronbach’s alpha was found to be 0.88, further confirming the tool’s reliability in this context.

### The Personal Social Capital Scale 16 (PSCS-16)

The Personal Social Capital Scale (PSCS) is a theoretical tool designed to assess an individual’s social capital, incorporating both bonding and bridging dimensions. In response to the need for concise instruments suitable for large-scale surveys, Wang et al. (47) developed two abbreviated versions: the PSCS-16 and PSCS-8, with 16 and 8 items, respectively (47). The PSCS-16 consists of 16 items, divided into two dimensions: bonding capital (8 items) and bridging capital (8 items). The scale uses two 5-point Likert-type response options: one assesses the “network size” with responses ranging from 1 (a few) to 5 (a lot), and the other evaluates “how many network members” are present, with responses from 1 (none) to 5 (all). A higher total score reflects a greater perceived level of personal social capital. According to Wang et al. (47), the PSCS-16 demonstrated strong validity and reliability (47). Exploratory Factor Analysis (EFA) revealed a two-factor structure, with significant factor loadings. Confirmatory Factor Analysis (CFA) supported this structure, with excellent fit indices: a Comparative Fit Index (CFI) of 0.95, Tucker-Lewis Index (TLI) of 0.94, and Root Mean Square Error of Approximation (RMSEA) of 0.05. The scale also showed high internal consistency, with a Cronbach’s alpha of 0.90, confirming its reliability and validity as a tool for assessing personal social capital. In our study, Cronbach’s alpha was 0.88.

## Study procedures

### Tool preparation and pilot study

The research instruments, including the DASS-21, PSCS-16, and SFS-8, were meticulously translated into Arabic by bilingual experts fluent in both English and Arabic to ensure precision and cultural relevance. To validate the translations, a back-translation process was implemented, ensuring linguistic equivalence and resolving any discrepancies between the original and translated versions. Following this, face validity assessments were conducted for each instrument, with expert panels reviewing the translated tools to ensure that they accurately represented the intended constructs within the Arabic cultural context. Additionally, feedback was gathered from potential participants to evaluate the clarity, relevance, and cultural appropriateness of the items, ensuring that the instruments were both understandable and relevant to the target population.

Reliability of the instruments was tested using statistical methods, including Cronbach’s alpha, to assess internal consistency. A pilot study was then carried out with a sample of 30 older adults to further evaluate the clarity, relevance, and reliability of the translated instruments. The participants for the pilot study were selected from a similar demographic but were excluded from the main study to prevent bias. During the pilot study, participants were asked to complete the instruments, and their responses were analyzed to identify any ambiguities or areas of confusion. Feedback from these participants was also solicited to ensure the items were culturally appropriate and easy to understand. Based on the results, no major modifications were required, confirming that the instruments were suitable for the main study. The pilot study provided valuable insights into the usability and reliability of the instruments, ensuring that the tools were appropriate for use in the target population of older adults in the main study.

### Data collection

The data collection process commenced with a comprehensive orientation session for participants, during which the study’s objectives were clearly outlined, and the voluntary nature of participation was emphasized. Researchers were readily available to address any inquiries or concerns, and confidentiality protocols were thoroughly explained to build trust. Conducted by trained researchers, data collection took place between February and April 2025. Before participation, all individuals provided written informed consent. To maintain anonymity, participants were instructed to exclude any personally identifiable details from the questionnaires. Participation remained entirely voluntary, with the option to withdraw at any time. The questionnaires were distributed in the clinic’s waiting areas, allowing participants to complete them at their convenience. Data collection was carried out from Saturday to Thursday, between 10 a.m. and 2 p.m., ensuring accessibility and a thorough data-gathering process.

### Ethical considerations

Ethical approval for this study was granted by the Research Ethics Committee of the Faculty of Nursing at Zagazig University, Egypt, under reference number (235). Permission to conduct the study was also obtained from the clinic directors, who were thoroughly briefed on the study’s objectives. The research adhered to the ethical standards outlined in the Declaration of Helsinki, ensuring the protection of participants’ rights and well-being. Written informed consent was obtained from all participants after they received a detailed explanation of the study’s purpose. Participants’ privacy and anonymity were rigorously maintained, with all data being handled confidentially. They were also informed of their right to withdraw from the study at any time without facing any consequences.

### Data analysis

Researcher analysis the data collected by SPSS 27.0 to examine the survey responses from the 316 participants (48). Frequencies (number and percentage) and mean ± standard deviations (S.D.) were used to summarize general characteristics of participants and for descriptive statistics. Multicollinearity among the independent variables was also evaluated using Variance Inflation Factor (VIF) and tolerance values. The results indicated no evidence of multicollinearity, as all VIF values were below the commonly accepted threshold of 10 and tolerance values were above 0.10. The correlations between study variables were evaluated by Spearman correlation analysis. Linear regression analysis tests are used to measure the influence of mental health and social capital on social frailty in older adults. *p*-value < 0.05 was deemed statistically significant, and *p*-value < 0.001 was deemed statistically extremely significant (49).

## Results

Table 1 shows that 49.4% of the participants were aged 60 to less than 65 years, 55.1% were female, and the majority were married. In addition, 47.5% had basic education, 66.5% were from rural areas, 40.2% were retired, 57.6% had sufficient income, and 83.2% did not smoke cigarettes. Furthermore, 81.3% did not engage in regular exercise, 67.1% were from extended families, 62.3% had a history of chronic disease, and 37.3% had a duration of 5 years or more since diagnosis with a chronic disease. Finally, 37% had never participated in social activities (e.g., clubs or community events).

**Table 1:** Personal and medical characteristics among participants (*n* = 316).

Personal data	Frequency	Percentage
Age		
From 60 to less than 65	156	49.4
From 65 to less than 70	78	24.7
70 years and above	82	25.9
Sex		
Male	142	44.9
Female	174	55.1
Marital status		
Single	22	7.0
Married	174	55.1
Divorced	17	5.4
Widow	103	32.6
Level of education		
Basic education	150	47.5
Secondary	51	16.1
University and above	115	36.4
Residence		
Urban	106	33.5
Rural	210	66.5
Job		
Self-employed	58	18.4
Retired	127	40.2
Housewife	103	32.6
Manual workers	8	2.5
Farmer	20	6.3
Income		
Enough	182	57.6
Not enough	103	32.6
Enough and save	31	9.8
Cigarette smoking		
Yes	53	16.8
No	263	83.2
Regular exercise		
Yes	59	18.7
No	257	81.3
Type of family		
Nuclear	104	32.9
Extended	212	67.1
History of chronic diseases		
Yes	197	62.3
No	119	37.7
Duration since diagnosis with a chronic disease		
No	110	34.8
Less than 5 years	88	27.8
5 years and more	118	37.3
How often do you participate in social activities (e.g., clubs, community events)?		
Daily	22	7.0
Weekly	29	9.2
Monthly	46	14.6
Rarely	102	32.3
Never	117	37.0

Table 2 reveals that the mean score of the participants’ depression anxiety stress scale was 46.28 ± 12.339, with its subscale as anxiety 12.68 ± 4.213, stress was 16.18 ± 4.710, and depression was 17.42 ± 5.191. Also, the mean score of the participants’ personal social capital scale was 29.96 ± 11.275, with its subscales as the bonding capital subscale 19.36 ± 6.155, and the bridging capital subscale was 10.68 ± 7.065. Also, the mean score of the participants’ social frailty scale was 5.55 ± 1.443.

**Table 2:** Descriptive analysis between study variables (*n* = 316).

Variables	N	Minimum	Maximum	Mean	Std. deviation
Depression Anxiety Stress Scale	316	0.00	63.00	46.28	12.339
Anxiety	316	0.00	21.00	12.68	4.213
Stress	316	0.00	21.00	16.18	4.710
Depression	316	0.00	21.00	17.42	5.191
Personal social capital scale	316	16.00	68.00	29.96	11.275
Bonding capital subscale	316	8.00	40.00	19.36	6.155
Bridging capital subscale	316	8.00	40.00	10.68	7.065
Social frailty scale	316	0.00	8.00	5.55	1.443

Table 3 the correlation analysis demonstrated several significant relationships among the study variables and their subdimensions. A strong positive correlation was found between the total Depression Anxiety Stress construct and social frailty (r = 0.659, *p* < 0.001), indicating that higher psychological distress is associated with increased social frailty among older adults. Regarding the psychological distress subdimensions, anxiety showed strong positive correlations with both stress (r = 0.621, *p* < 0.001) and depression (r = 0.608, *p* < 0.001). Similarly, stress and depression were strongly correlated (r = 0.612, *p* < 0.001), confirming the internal coherence of the distress construct. In contrast, personal social capital was strongly and negatively correlated with overall psychological distress (r = −0.816, *p* < 0.001) and social frailty (r = −0.618, *p* < 0.001), indicating that higher levels of social capital are associated with lower psychological distress and reduced social frailty. However, when examining the subdimensions of social capital, neither bonding social capital nor bridging social capital showed statistically significant correlations with depression, anxiety, stress, or social frailty (*p* > 0.05). The only significant association observed at the subdimension level was a moderate positive correlation between bonding and bridging capital (r = 0.511, *p* < 0.001).

**Table 3:** Correlation between study variables among participants (*n* = 316).

Variables	1	2	3	4	5	6	7	8
Depression anxiety stress	1.000							
	.							
Anxiety	0.059	1.000						
	0.297	.						
Stress	−0.004-	0.621^**^	1.000					
	0.949	0.000	.					
Depression	0.013	0.608^**^	0.612^**^	1.000				
	0.819	0.000	0.000	.				
Personal social capital	−0.816^**^	0.028	−0.039-	−0.056-	1.000			
	0.000	0.615	0.486	0.320	.			
Bonding capital	0.052	0.087	0.050	0.021	0.061	1.000		
	0.355	0.121	0.376	0.709	0.277	.		
Bridging capital	−0.033-	0.039	0.009	0.010	−0.036-	0.511^**^	1.000	
	0.560	0.489	0.879	0.866	0.520	0.000	.	
Social frailty	0.659^**^	0.078	0.012	0.063	−0.618^**^	0.092	0.054	1.000
	0.000	0.167	0.836	0.265	0.000	0.102	0.335	.

r = Spearman Correlation, ** Correlation is highly significant at the 0.01 level (2-tailed).

Table 4 a multiple linear regression analysis was conducted to identify predictors of social frailty among older adults. The overall model was statistically significant (*F* = 24.510, *p* < 0.001), explaining 13.5% of the variance in social frailty (R^2^ = 0.135, Adjusted R^2^ = 0.130), indicating a modest explanatory power. Among the main study variables, the Depression Anxiety Stress Scale was a significant positive predictor of social frailty (B = 0.320, *β* = 0.369, t = 4.215, *p* < 0.001), suggesting that higher levels of psychological distress are associated with increased social frailty. The Personal Social Capital Scale was a significant negative predictor (B = −0.229, β = −0.327, t = −4.975, *p* < 0.001), indicating that higher social capital is associated with lower social frailty. However, when examining the subdimensions of social capital, neither bonding capital (B = 1.422, β = 0.073, *p* = 0.248) nor bridging capital (B = 2.643, β = 0.084, *p* = 0.303) were statistically significant predictors (*p* > 0.05). Regarding socio-demographic and health-related variables: Income was a significant predictor (B = −8.563, β = −0.301, *p* < 0.001), indicating that lower income is associated with higher social frailty, Regular exercise was a significant negative predictor (B = −1.236, β = −0.156, *p* = 0.002), History of chronic disease significantly increased social frailty (B = 3.434, β = 0.180, *p* < 0.001), Longer duration of chronic disease was also significant (B = 6.452, β = 0.120, *p* = 0.014), Participation in social activities was a significant protective factor (B = −0.297, β = −0.156, *p* = 0.001).

**Table 4:** Linear regression model of predictor factors for social frailty scale among older adults (*n* = 316).

Model	Unstandardized Coefficients		Standardized Coefficients	t	Sig.	95.0% Confidence Interval for B	
	B	Std. Error	Β			Lower Bound	Upper Bound
(Constant)	4.061	0.269		15.085	0.000	3.531	4.590
Depression Anxiety Stress Scale	0.320	0.009	0.369	4.215	**<0.001**	0.302	0.338
Personal social capital scale	−0.229	0.010	0.327	−4.975	**<0.001**	0.209	0.249
Bonding capital	1.422	1.046	0.073	1.359	0.248	−0.543	0.546
Bridging capital	2.643	1.633	0.084	1.724	0.303	−0.56	6.35
Age	0.246	1.415	0.008	0.176	0.863	2.54	3.03
Sex	2.845	1.733	0.094	1.636	0.103	0.578	6.260
Marital status	2.356	2.375	0.046	0.991	0.321	−2.308	7.014
Level of education	4.017	2.222	0.098	1.857	0.072	−0.355	8.393
Residence	1.522	1.056	0.083	1.459	0.148	−0.593	0.544
Job	2.843	1.733	0.094	1.624	0.103	−0.58	6.26
Income	−8.563	1.556	0.301	−5.531	**<0.001**	5.50	11.62
Cigarette smoking	1.141	3.403	0.017	0.331	0.736	−5.55	7.83
Regular exercise	−1.236	0.391	0.156	−3.163	**0.002**	0.47	2.00
Type of family	1.182	2.261	0.025	0.524	0.601	−3.28	5.65
History of chronic diseases	3.434	0.933	0.180	3.653	**<0.001**	1.61	5.25
Duration since diagnosis with a chronic disease	6.452	2.621	0.120	2.482	**0.014**	1.338	11.567
Participate in social activities	−0.297	0.083	0.156	−3.305	**0.001**	0.120	0.471
F	24.510						
P	<0.001						
R	0.368						
R square	0.135						
Adjusted R square	0.130						

B = unstandardized coefficient β = standardized coefficient. Bold at *p* < 0.01.

## Discussion

This study examined the influence of mental health and social capital on social frailty among older adults in Egypt, highlighting the crucial role of psychosocial factors in determining vulnerability in later life. Findings indicated that a considerable proportion of participants experienced chronic diseases, limited physical activity, and reduced social engagement, all of which are well-established contributors to social frailty (5, 7). Additionally, participants reported high psychological distress and elevated social frailty scores, suggesting that a significant proportion of the sample was socially vulnerable. These findings are consistent with global reports indicating an increase in mental health issues and social disconnection among older adults, especially in low-resource settings (1, 50, 51). The study findings further suggest that the sample represents a medically and socially vulnerable group, characterized by high prevalence of chronic illness, low engagement in health-promoting behaviors, and limited participation in social activities, which may collectively exacerbate the risk of social frailty through both biological and social pathways.

Social capital levels were moderate, with bonding capital exceeding bridging capital, reflecting the structure of social relationships in collectivist contexts (40, 52). However, regression results indicated that neither bonding nor bridging social capital had a statistically significant independent association with social frailty. This finding contrasts with several previous studies that reported differential effects of bonding and bridging capital (11, 53) and may suggest that the influence of these subdimensions is context-dependent or mediated by other factors such as health status or socioeconomic conditions. One possible explanation is that bonding and bridging capital may operate synergistically rather than independently, and when included simultaneously in the model, their unique contributions become attenuated due to shared variance (54, 55).

In contrast, overall social capital demonstrated a significant inverse association with social frailty. These finding highlights that the cumulative or global perception of social connectedness may be more relevant than specific structural components of social networks. It is possible that the protective effects of social capital emerge through integrated mechanisms, including emotional support, access to resources, and reinforcement of social identity, which are not fully captured when dimensions are analyzed separately (27, 50).

Psychological distress was identified as a strong predictor of social frailty.

Specifically, the total score of the Depression Anxiety Stress Scale showed a robust positive association with social frailty, indicating that higher levels of distress significantly increase vulnerability. This aligns with prior research demonstrating that psychological distress contributes to frailty through both behavioral and physiological mechanisms (6, 56). From a behavioral perspective, individuals experiencing distress are more likely to withdraw from social interactions, reduce participation in daily activities, and experience diminished motivation, all of which directly contribute to social frailty. From a biological perspective, chronic psychological distress is associated with dysregulation of the HPA axis and increased inflammatory responses, which may accelerate functional decline and frailty progression (9, 51).

Although the study conceptually included subdimensions of psychological distress (depression, anxiety, and stress), the regression model relied on the total score. Therefore, it was not possible to determine which specific component had the strongest effect. Given evidence that depression is often more strongly associated with frailty than anxiety or stress, future studies should disaggregate these components to provide more precise insights (23, 26). A significant inverse association between social capital and social frailty supports the buffering hypothesis, whereby social resources mitigate the negative effects of stress and adversity (12, 57). However, the absence of significant effects for bonding and bridging capital individually suggests that the buffering mechanism may operate at a more holistic level, rather than through specific types of ties (58).

Importantly, interaction effects between psychological distress and social capital were not tested in the present study, as the regression model did not include an interaction term. While prior research suggests that social capital may moderate the relationship between stress and health outcomes (59), the current findings cannot confirm this mechanism. In addition to psychosocial factors, several structural determinants were significantly associated with social frailty. Higher income was associated with lower social frailty, suggesting that economic resources may enhance access to social participation opportunities and healthcare services. Similarly, regular exercise and participation in social activities were protective, likely due to their dual role in maintaining both physical function and social engagement (23, 60). In contrast, chronic diseases and longer disease duration were significant risk factors, reflecting the cumulative burden of illness on functional capacity and social integration. These findings are consistent with multidimensional models of frailty, which emphasize the interplay between physical, psychological, and social domains (61, 62).

Furthermore, Social Production Functions Theory (SPFT) offers a valuable framework for interpreting these results. According to SPFT, individuals strive to fulfill basic social needs such as affection, behavioral confirmation, and status through social structures (18, 19). When these structures are disrupted by life events like retirement, bereavement, or the migration of children, older adults may experience social disengagement and frailty. This theoretical lens helps explain how deficits in social capital can precipitate a downward trajectory in psychological and social well-being (16, 36, 54).

Interestingly, several demographic variables such as age, sex, education, and marital status were not significant predictors in the adjusted model. This may indicate that their effects are indirect, operating through mediating variables such as health status, income, or social participation, rather than exerting direct influence on social frailty (63). Taken together, the findings suggest that social frailty is a multifactorial construct shaped by complex interactions between psychological distress, social resources, and health-related factors. The stronger effect of psychological distress relative to social capital in the regression model underscores the central role of mental health in driving social vulnerability in this population (21, 28).

The combined influence of mental health and social capital on social frailty represents an important but still underexplored domain in gerontology. While the present study did not statistically test an interaction effect between these variables, the findings clearly demonstrate that both poor mental health and low social capital contribute to increased social frailty. These results suggest that older adults experiencing psychological distress alongside limited social resources may be at greater risk of social vulnerability. This co-occurrence highlights a potentially reinforcing relationship between the two constructions, even though their statistical interaction was not examined in the current analysis (4, 54, 55).

Consistent with previous literature, mental health and social capital are often interrelated, where diminished psychological well-being may reduce individuals’ ability to build or maintain social networks, while limited social capital may exacerbate feelings of distress and isolation (33, 58). Therefore, rather than interpreting these variables as interacting predictors, the current findings support their conceptual interdependence and cumulative impact on social frailty.

Succinctly, the current study offers a comprehensive analysis of how mental health and social capital collectively influence social frailty in older adults. By integrating psychosocial variables into the frailty framework, it aligns with the broader shift in aging research toward a multidimensional model of vulnerability. The results affirm that psychological distress and reduced social capital are significant, culturally sensitive indicators of social frailty, and that their effects are independently significant yet conceptually interconnected. These insights contribute meaningfully to gerontological literature and underscore the need to reconceptualize frailty as an integrative phenomenon shaped by social, psychological, and physical dimensions.

## Limitations

While this study offers important insights into psychosocial determinants of social frailty, several limitations warrant consideration. The cross-sectional design, while valuable for establishing initial associations, prevents definitive causal conclusions about the relationships between mental health, social capital, and social frailty. The likely bidirectional nature of these relationships, where social frailty may both result from and contribute to psychological distress and diminishing social capital, requires longitudinal investigation to unravel temporal sequences and causal mechanisms. The convenience sampling approach constrains the generalizability of findings from a single tertiary care setting, which may not adequately represent the broader Egyptian older adult population, particularly those with different healthcare utilization patterns or residing in institutional settings. Future studies would benefit from employing stratified random sampling across diverse community settings, including primary care centers and rural areas, to enhance representativeness.

Methodologically, the exclusive reliance on self-report measures introduces potential biases, including social desirability effects in reporting psychological symptoms and possible inaccuracies in recalling social engagement patterns. Future research could strengthen methodological rigor through data triangulation, incorporating clinical interviews, informant reports, and objective behavioral measures of social participation. Furthermore, the modest proportion of variance explained in our regression model suggests significant unmeasured contributors to social frailty. Subsequent investigations should include a comprehensive assessment of potential confounders and mediators, including objective physical health indicators, detailed cognitive functioning beyond basic screening, functional mobility measures, and qualitative aspects of social relationships. Integrating these elements through advanced statistical modeling could substantially enhance our understanding of the multifactorial nature of social frailty and inform more effective, targeted interventions for vulnerable older populations.

## Conclusion

This study provides empirical evidence that psychological distress and diminished social capital are significant, independent predictors of social frailty among older adults in Egypt. The findings affirm that social frailty is a multidimensional construct influenced by distinct yet interconnected psychosocial domains. The positive association between mental health symptomatology and social frailty underscores the role of internal psychological states in eroding social functioning. Conversely, the robust negative association with social capital highlights the protective capacity of robust social networks and resources in preserving an individual’s social integrity.

These results necessitate a paradigm shift in geriatric assessment and intervention strategies. Moving beyond a purely biomedical model, a holistic approach that integrates mental health screening with the evaluation of social resource deficits is crucial for early identification of at-risk individuals. Future interventions should be dual-pronged, aiming both to mitigate psychological distress through targeted support and to actively build bridging and bonding social capital through community-based programs.

### Nursing implications

The findings underscore the imperative for nurses to integrate standardized assessments for social frailty, psychological distress, and social capital into routine geriatric practice, enabling early identification of at-risk older adults. This necessitates a shift towards holistic care planning, where nurses develop targeted interventions, provide education on maintaining social networks, and facilitate referrals to mental health services and community resources. Furthermore, these results highlight the need to incorporate these concepts into nursing curricula and to champion interprofessional collaboration. Ultimately, nurses are pivotal in leading the development and implementation of psychosocial strategies that mitigate social frailty, thereby promoting resilience and overall well-being in aging populations.

## Data Availability

The raw data supporting the conclusions of this article will be made available by the authors, without undue reservation.
